# Obituary

**Published:** 1893-07

**Authors:** 


### Obituary.

DIED—At the home of his mother in Colfax, Ohio, Dr. Arthur St. Clair Graham, D.D.S., aged thirty-seven years and three months. That fell destroyer, consumption, was the occasion of his death.

Dr. Graham graduated in the Dental Department of the University of Michigan, in 1883, and practiced his profession first in Nelsonville, then in Pataskala, then for a time in Columbus, O., and subsequently in Asheville, N. C. In each of these places he was successful in his practice, and only changed in the hope of improving his health. His disease, however, continued to progress until it carried him down. Dr. Graham was a Christian gentleman of a high type of honor and integrity; the profession can ill afford to lose such men. We feel fully warranted in extending to his mother and the beloved friends of his family the warm-hearted sympathy of all who knew him.

DIED—June 14th, 1893, at her home in Jackson, Michigan, Mrs. Robinson, wife of Dr. J. A. Robinson, D.D.S.

Mrs. Robinson was about eighty-two years of age; had been quite an invalid for over two years; through all her weakness and suffering she was ever cheerful and resigned to her condition. She had been a lovely and beloved companion of him who is so extensively and favorably known throughout the dental profession for well nigh sixty years. She once possessed a vigorous constitution, but through great activity and many cares of life the machinery was simply worn out; this was the cause of the dissolution rather than the operation of any active disease. A great measure of loving sympathy will go out to the bereaved husband from all in the profession who know him, and there are hosts of them. May he and the loving sons and daughters be sustained and strengthened in this their great bereavement.

